# Supplementary material for: Conformational equilibrium shift underlies altered K+ channel gating as revealed by NMR
Source: Nat Commun. 2020 Oct 14;11:5168. doi: 10.1038/s41467-020-19005-3 (PMC7560842; doi:10.1038/s41467-020-19005-3)
Supplement: Supplementary file 1 — Supplementary information [file 41467_2020_19005_MOESM1_ESM.pdf]

Supplementary Information

Conformational equilibrium shift underlies altered K^+ channel gating as revealed by NMR

Iwahashi *et al.*

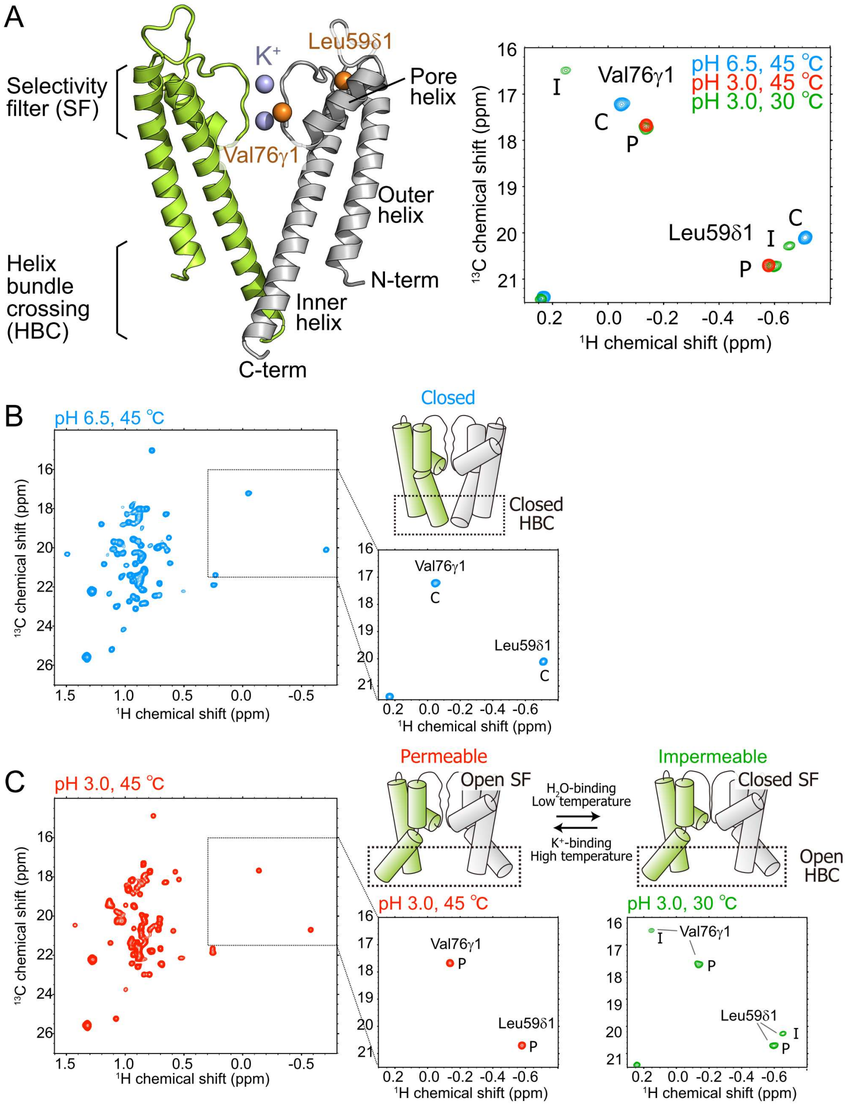

Supplementary Fig. 1 Three-state model of KcsA and NMR spectra of KcsA measured under different conditions. (A) Crystal structure of KcsA with the closed HBC gate (PDB ID: 1K4C). Only two facing subunits are shown for clarity. The methyl carbons of Leu59δ1 and Val76γ1 are shown as orange spheres (left). Overlay of the ^{1}H-^{13}C HMQC spectra of KcsA measured under different conditions (blue: pH 6.5 and 45 °C, red: pH 3.0 and 45 °C, green: pH 3.0 and 30 °C). The signals representing the impermeable, closed, and permeable states are denoted as I, C, and P, respectively. (B) ^{1}H-^{13}C HMQC spectrum of KcsA measured at pH 6.5 and 45 °C, in which KcsA mainly adopts the C state. The schematic model of the C state is shown above. (C) ^{1}H-^{13}C HMQC spectrum of KcsA measured at pH 3.0 and 45 °C, in which KcsA mainly adopts the P state (red). The regions for Leu59 and Val76 measured at pH 3.0 and 45 °C (red) and at pH 3.0 and 30 °C (green) are compared. At 30 °C, KcsA exists in an equilibrium between the P and I states. The schematic model of the equilibrium between the P and I states is shown above.

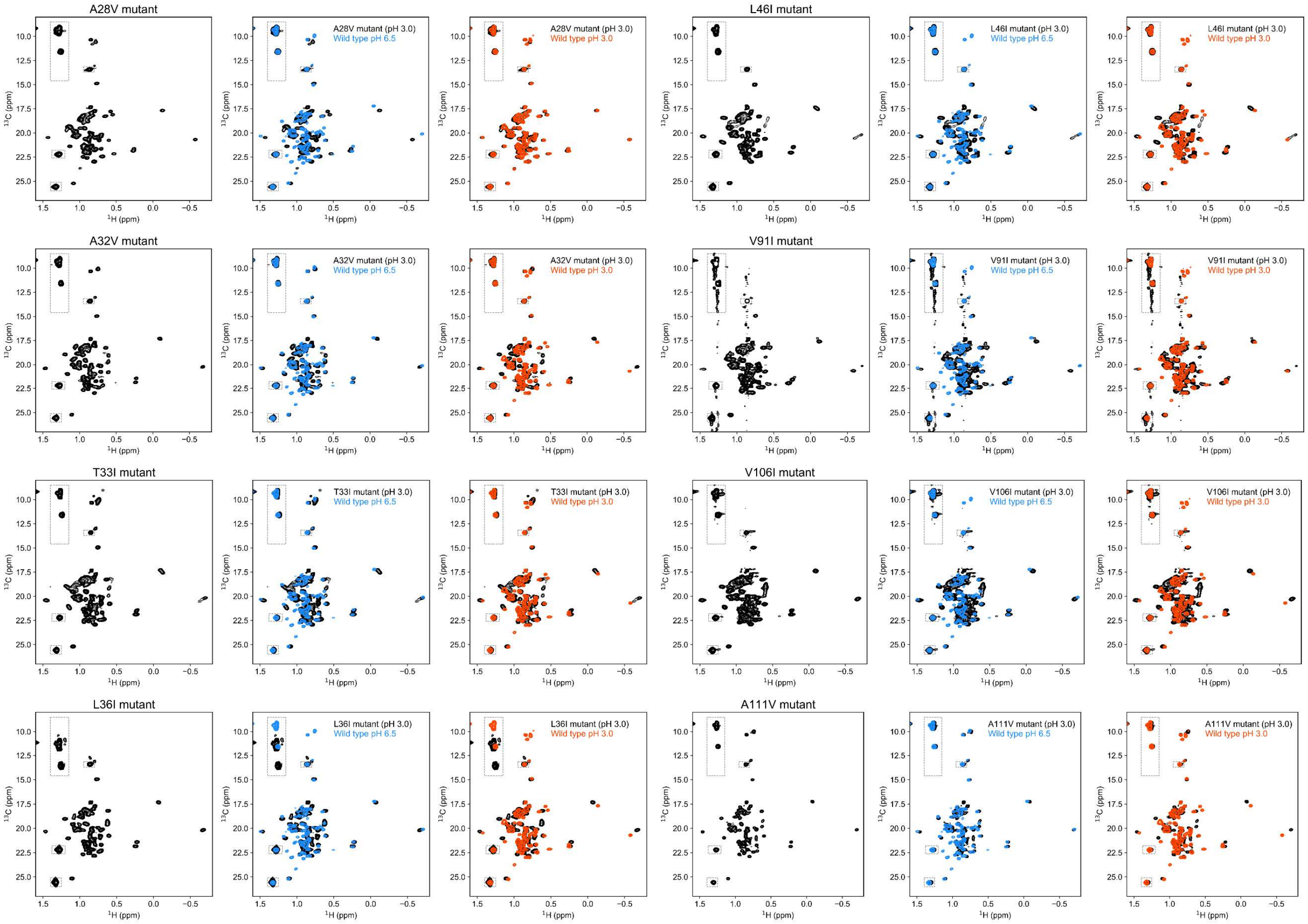

Supplementary Fig. 2 NMR spectra of KcsA mutants. ^{1}H-^{13}C HMQC spectra of KcsA mutants (A28V, A32V, T33I, L36I, L46I, V91I, V106I, and A111V) are shown. An overlay of the ^{1}H-^{13}C HMQC spectra of the wild type KcsA measured under different conditions (blue: pH 6.5 and 45 °C, red: pH 3.0 and 45 °C, in the presence of 100 mM KCl) is also shown. The Ileδ1, Leuδ1/2, and Valγ1/2 methyl groups were selectively labeled with ^{1}H and ^{13}C in the wild type and the A28V, A32V, T33I, and A111V mutants, and the Leuδ1/2 and Valγ1/2 methyl groups were labeled in the L36I, L46I, V91I, and V106I mutants, in an otherwise highly deuterated background. The spectra were recorded at pH 3.0 and 45 °C in the presence of 100 mM KCl, at 11.7 Tesla (500 MHz ^{1}H frequency for L36I), 14.1 Tesla (600 MHz ^{1}H frequency, for A28V, A32V, and L46I), or 18.8 Tesla (800 MHz ^{1}H frequency, for wild type, T33I, V91I, V106I, and A111V).

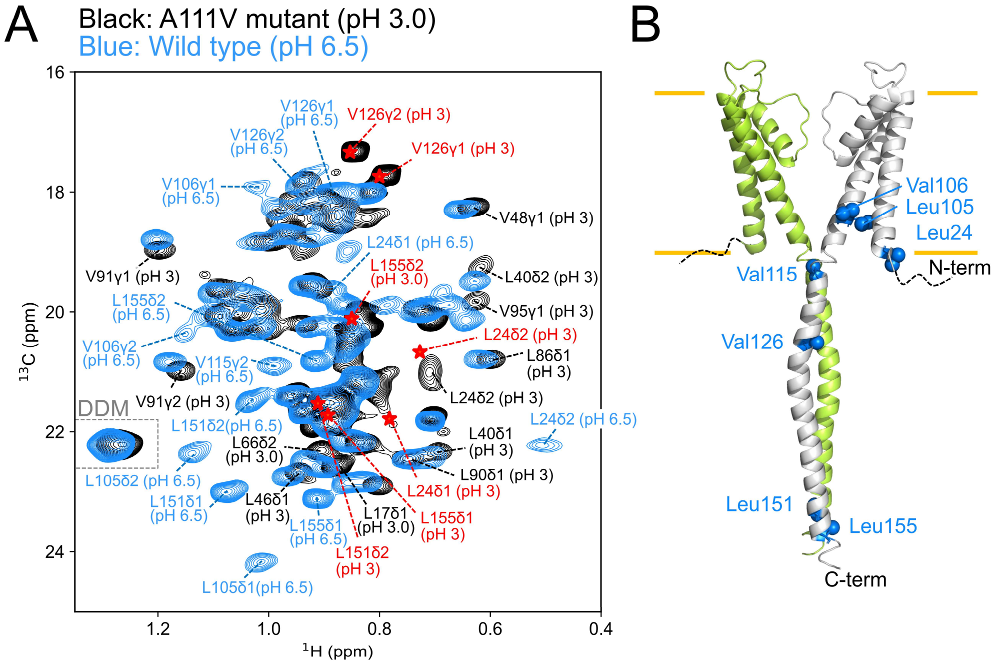

Supplementary Fig. 3 Enlarged view of the Leu/Val region of the ^{1}H-^{13}C HMQC spectra of the wild type and the A111V mutant. (A) Enlarged view of the Leu/Val region of overlaid ^{1}H-^{13}C HMQC spectra of the wild type at pH 6.5 (blue) and the A111V mutant at pH 3.0 (black). The spectra were measured at 45 °C and 18.8 Tesla (800 MHz ^{1}H frequency), in the presence of 100 mM KCl. Representative residues observed in the well-resolved region are labeled (black: the A111V mutant at pH 3.0, blue: wild type at pH 6.5, red: wild type at pH 3.0). The chemical shifts of Leu24, Val126, Leu151, and Leu155 of the wild type at pH 3.0 are indicated as red stars. (B) Mapping of the highlighted residues on the crystal structure of the full-length KcsA in the closed state (PDB ID: 3EFF)[61]. Only two facing subunits are shown for clarity.

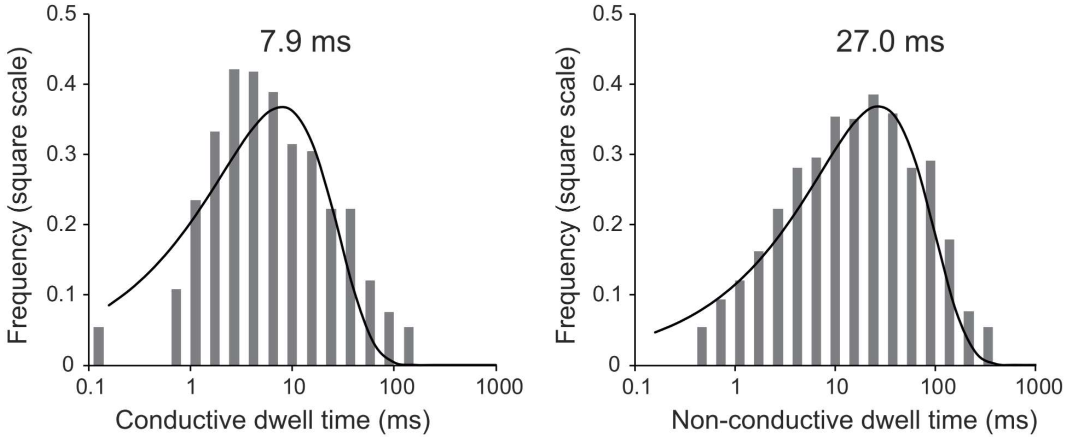

Supplementary Fig. 4 Conductive and non-conductive dwell time distributions in the single-channel recording of the V91I mutant. The distributions of the conductive (left) and non-conductive (right) dwell times are shown. The single-channel recording of the V91I mutant was analyzed. The lines denote the fitted probability density functions. The values of the mean conductive and non-conductive dwell times are shown. Source data are provided as a Source Data File.

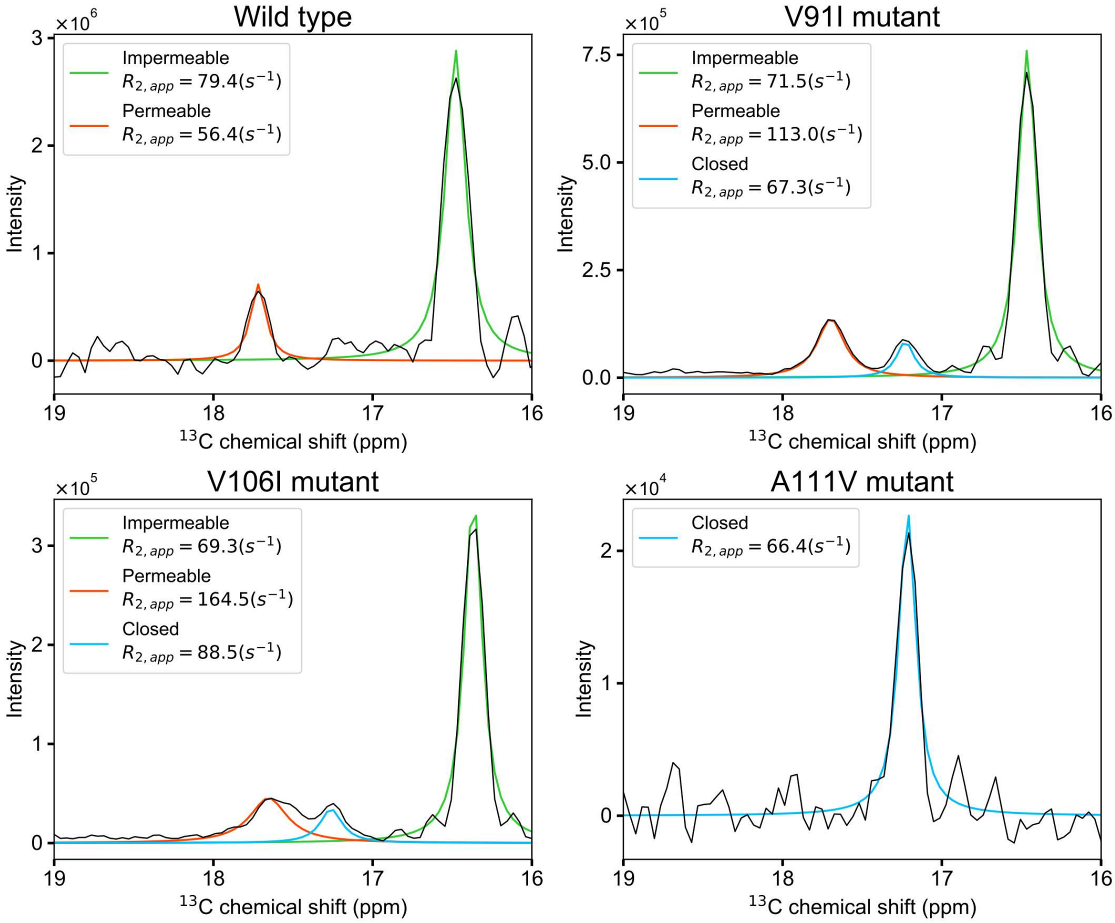

Supplementary Fig. 5 ^{13}C 1D projections of the V76γ1 region (^{1}H chemical shift range -0.176 to 0.213 ppm) of the wild type KcsA and its mutants. The spectra were recorded at 18.8 Tesla (^{1}H frequency 800 MHz), pH 3.0, and 25 °C. The exponential window function (line broadening factor 5 Hz) was applied for the ^{13}C dimension. We fitted the signal line shapes using the Lorentzian function and obtained the linewidths at half height. For the wild type, the impermeable (green) and permeable (red) signals were assumed. For the V91I and V106I mutants, impermeable (green), permeable (red), and closed (blue) signals were assumed. For the A111V mutant, the closed signal (blue) was assumed. The apparent transverse relaxation rates during the t_1 period, $R_{2,\text{ app}}$, were estimated from the fitted linewidths at half-height, taking the applied line-broadening factor into account.

Supplementary Table 1 KcsA DNA and primer sequences

	**DNA sequence (5'-3')**
KcsA (1-160)	ATGCCGCCGATGCTGTCCGGTCTTCTGGCGCGTCTGGTGAA<br>ACTGCTGCTGGGTCGCCACGGTTCCGCGCTGCACTGGCGTG<br>CAGCAGGTGCAGCAACCGTGCTGCTGGTGATCGTGCTGCTG<br>GCGGGTTCCTACCTGGCCGTGCTGGCGGAACGCGGTGCACC<br>GGGTGCGCAGCTGATCACCTATCCGCGTGCGCTGTGGTGGT<br>CCGTGGAAACCGCGACCACCGTGGGTTACGGTGACCTGTAC<br>CCGGTGACCCTGTGGGGTCGTCTGGTGGCGGTGGTGGTGAT<br>GGTGGCGGGTATCACCTCCTTCGGTCTGGTGACCGCGGCGC<br>TGGCGACCTGGTTCGTGGGTCGTGAACAAGAACGCCGTGG<br>TCACTTCGTGCGCCACTCCGAAAAAGCGGCGGAAGAAGCG<br>TACACCCGTACCACCCGTGCGCTGCACGAACGTTTCGACCG<br>TCTGGAACGTATGCTGGACGACAACCGCCGT
A28V	Forward: 5' CTGCACTGGCGTGTAGCAGGTGCAGC 3'<br>Reverse: 5' GCTGCACCTGCTACACGCCAGTGCAG 3
A32V	Forward: 5' GCAGCAGGTGCAGTAACCGTGCTGCTGG 3'<br>Reverse: 5' CCAGCAGCACGGTTACTGCACCTGCTGC 3
V34I	Forward: 5' GCAGGTGCAGCAACCATCCTGCTGGTGATCGTG 3'<br>Reverse: 5' CACGATCACCAGCAGGATGGTTGCTGCACCTGC 3'
L35I	Forward: 5' CAGGTGCAGCAACCGTGATCCTGGTGATCGTGCTGC 3'<br>Reverse: 5' GCAGCACGATCACCAGGATCACGGTTGCTGCACCTG 3'
L36I	Forward: 5' GCAGCAACCGTGCTGATCGTGATCGTGCTGCTG 3'<br>Reverse: 5' CAGCAGCACGATCACGATCAGCACGGTTGCTGC 3'
V37I	Forward: 5' CAACCGTGCTGCTGATCATCGTGCTGCTGGCG 3'<br>Reverse: 5' CGCCAGCAGCACGATGATCAGCAGCACGGTTG 3'
V39I	Forward: 5' GTGCTGCTGGTGATCATCCTGCTGGCGGGTTC 3'<br>Reverse: 5' GAACCCGCCAGCAGGATGATCACCAGCAGCAC 3'
L40I	Forward: 5' CTGCTGGTGATCGTGATCCTGGCGGGTTCCTAC 3'<br>Reverse: 5' GTAGGAACCCGCCAGGATCACGATCACCAGCAG 3'
L41I	Forward: 5' GCTGGTGATCGTGCTGATCGCGGGTTCCTACCTGGC 3'<br>Reverse: 5' GCCAGGTAGGAACCCGCGATCAGCACGATCACCAGC 3'
L46I	Forward: 5' GGCGGGTTCCTACATCGCCGTGCTGGCGG 3'<br>Reverse: 5' CCGCCAGCACGGCGATGTAGGAACCCGCC 3'
V48I	Forward: 5' GTTCCTACCTGGCCATCCTGGCGGAACGCGG 3'<br>Reverse: 5' CCGCGTTCCGCCAGGATGGCCAGGTAGGAAC 3'
L49I	Forward: 5' CCTACCTGGCCGTGATCGCGGAACGCGGTG 3'<br>Reverse: 5' CACCGCGTTCCGCGATCACGGCCAGGTAGG 3'

L66I	Forward: 5' CCTATCCGCGTGCGATCTGGTGGTCCGTGG 3'<br>Reverse: 5' CCACGGACCACCAGATCGCACGCGGATAGG 3'
V70I	Forward: 5' CGCTGTGGTGGTCCATCGAAACCGCGACCAC 3'<br>Reverse: 5' GTGGTCGCGGTTTCGATGGACCACCACAGCG 3'
L81I	Forward: 5' CGTGGGTTACGGTGACATCTACCCGGTGACCCTG 3'<br>Reverse: 5' CAGGGTCACCGGGTAGATGTCACCGTAACCCACG 3'
V84I	Forward: 5' GTGACCTGTACCCGATCACCCTGTGGGGTCG 3'<br>Reverse: 5' CGACCCCACAGGGTGATCGGGTACAGGTCAC 3'
L86I	Forward: 5' CTGTACCCGGTGACCATCTGGGGTCGTCTGGTG 3'<br>Reverse: 5' CACCAGACGACCCCAGATGGTCACCGGGTACAG 3'
V91I	Forward: 5' CTGTGGGGTCGTCTGATCGCGGTGGTGGTGATG 3'<br>Reverse: 5' CATCACCACCACCGCGATCAGACGACCCCACAG 3'
V93I	Forward: 5' GTCGTCTGGTGGCGATCGTGGTGATGGTGGC 3'<br>Reverse: 5' GCCACCATCACCACGATCGCCACCAGACGAC 3'
V94I	Forward: 5' CGTCTGGTGGCGGTGATCGTGATGGTGGCGGG 3'<br>Reverse: 5' CCCGCCACCATCACGATCACCGCCACCAGACG 3'
V95I	Forward: 5' CTGGTGGCGGTGGTGATCATGGTGGCGGGTATC 3'<br>Reverse: 5' GATACCCGCCACCATGATCACCACCGCCACCAG 3'
V97I	Forward: 5' CGGTGGTGGTGATGATCGCGGGTATCACCTCC 3'<br>Reverse: 5' GGAGGTGATACCCGCGATCATCACCACCACCG 3'
L105I	Forward: 5' CACCTCCTTCGGTATCGTGACCGCGGCGC 3'<br>Reverse: 5' GCGCCGCGGTCACGATACCGAAGGAGGTG 3'
V106I	Forward: 5' CTCCTTCGGTCTGATCACCGCGGCGCTGG 3'<br>Reverse: 5' CCAGCGCCGCGGTGATCAGACCGAAGGAG 3'
L110I	Forward: 5' GGTGACCGCGGCGATCGCGACCTGGTTCG 3'<br>Reverse: 5' CGAACCAGGTCGCGATCGCCGCGGTCACC 3'
A111V	Forward: 5' CCGCGGCGCTGGTGACCTGGTTCG 3'<br>Reverse: 5' CGAACCAGGTCACCAGCGCCGCGG 3'
V115I	Forward: 5' GCTGGCGACCTGGTTCATCGGTCGTGAACAAGAAC 3'<br>Reverse: 5' GTTCTTGTTCACGACCGATGAACCAGGTCGCCAGC 3'
