# Supplementary material for: Conformational equilibrium shift underlies altered K+ channel gating as revealed by NMR
Source: Nat Commun. 2020 Oct 14;11:5168. doi: 10.1038/s41467-020-19005-3 (PMC7560842; doi:10.1038/s41467-020-19005-3)
Supplement: Supplementary file 3 — Reporting summary [file 41467_2020_19005_MOESM3_ESM.pdf]

nature research

Corresponding author(s): Ichio Shimada

Last updated by author(s): Sep 10, 2020

Reporting Summary

Nature Research wishes to improve the reproducibility of the work that we publish. This form provides structure for consistency and transparency in reporting. For further information on Nature Research policies, see our Editorial Policies and the Editorial Policy Checklist.

Statistics

For all statistical analyses, confirm that the following items are present in the figure legend, table legend, main text, or Methods section.

n/a	Confirmed	
☐	☒	The exact sample size (*n*) for each experimental group/condition, given as a discrete number and unit of measurement
☐	☒	A statement on whether measurements were taken from distinct samples or whether the same sample was measured repeatedly
☒	☐	The statistical test(s) used AND whether they are one- or two-sided<br>*Only common tests should be described solely by name; describe more complex techniques in the Methods section.*
☒	☐	A description of all covariates tested
☒	☐	A description of any assumptions or corrections, such as tests of normality and adjustment for multiple comparisons
☐	☒	A full description of the statistical parameters including central tendency (e.g. means) or other basic estimates (e.g. regression coefficient) AND variation (e.g. standard deviation) or associated estimates of uncertainty (e.g. confidence intervals)
☒	☐	For null hypothesis testing, the test statistic (e.g. *F*, *t*, *r*) with confidence intervals, effect sizes, degrees of freedom and *P* value noted<br>*Give P values as exact values whenever suitable.*
☒	☐	For Bayesian analysis, information on the choice of priors and Markov chain Monte Carlo settings
☒	☐	For hierarchical and complex designs, identification of the appropriate level for tests and full reporting of outcomes
☒	☐	Estimates of effect sizes (e.g. Cohen's *d*, Pearson's *r*), indicating how they were calculated

Our web collection on statistics for biologists contains articles on many of the points above.

Software and code

Policy information about availability of computer code

Data collection	TopSpin 2.1 or 3.1 (Bruker Biospin) for NMR data collection. UCSF Sparky version 3.114 (T. D. Goddard and D. G. Kneller, Sparky 3, University of California, San Francisco, CA) for visualizing NMR spectra.
Data analysis	TopSpin 2.1 or 3.1 (Bruker Biospin) for NMR data processing. Port-a-Patch system (Nanion Technologies) and QuB version 0.0.1 (https://qub.mandelics.com/) for single-channel analyses. The structures were rendered using PyMOL version 2.4.0. Codes used to fit the single-channel all points histograms and NMR line-shapes are available from the corresponding author upon request.

For manuscripts utilizing custom algorithms or software that are central to the research but not yet described in published literature, software must be made available to editors and reviewers. We strongly encourage code deposition in a community repository (e.g. GitHub). See the Nature Research guidelines for submitting code & software for further information.

Data

Policy information about availability of data

All manuscripts must include a data availability statement. This statement should provide the following information, where applicable:

- Accession codes, unique identifiers, or web links for publicly available datasets
- A list of figures that have associated raw data
- A description of any restrictions on data availability

Sequence information on Streptomyces lividans KcsA, Homo sapiens Kv1.1, and Drosophila melanogaster Shaker Kv channels are available in the UniProt Knowledgebase under accession codes P0A334, Q09470, and P08510. The PDB accession codes 1K4C, 3EFF, and 5VK6 were used in this study. The source data underlying Fig. 4 and Supplementary Fig. 4 are provided as a Source Data file. Data pertaining to this study are contained within the paper and its Supplementary Inforamation, or are available from the corresponding author upon reasonable request.

Field-specific reporting

Please select the one below that is the best fit for your research. If you are not sure, read the appropriate sections before making your selection.

☒ Life sciences ☐ Behavioural & social sciences ☐ Ecological, evolutionary & environmental sciences

For a reference copy of the document with all sections, see nature.com/documents/nr-reporting-summary-flat.pdf

Life sciences study design

All studies must disclose on these points even when the disclosure is negative.

Sample size	No statistical methods were used to predetermine sample sizes. For all data sets provided in this work, we chose replicate sizes according to standard methods provided in the literature and to our own preliminary experiments so as to provide sufficient power for statistical comparison (where applicable).
Data exclusions	The data were not excluded from the analyses.
Replication	All attempts at replication were successful. The numbers of experiments conducted are described in the Methods section, where applicable.
Randomization	Randomization method was not utilized. This was not a clinical trial (i.e., no animals or humans were involved) and different bacterial strains were not being compared to each other.
Blinding	Investigator was not blinded to the group allocation. This was not a clinical trial (i.e., no animals or humans were involved) and group allocation was not a part of experimental design.

Reporting for specific materials, systems and methods

We require information from authors about some types of materials, experimental systems and methods used in many studies. Here, indicate whether each material, system or method listed is relevant to your study. If you are not sure if a list item applies to your research, read the appropriate section before selecting a response.

Materials & experimental systems

n/a	Involved in the study	
☒	☐	Antibodies
☒	☐	Eukaryotic cell lines
☒	☐	Palaeontology and archaeology
☒	☐	Animals and other organisms
☒	☐	Human research participants
☒	☐	Clinical data
☒	☐	Dual use research of concern

Methods

n/a	Involved in the study	
☒	☐	ChIP-seq
☒	☐	Flow cytometry
☒	☐	MRI-based neuroimaging
